# New Ethical Issues in Cerebral Palsy

**DOI:** 10.3389/fneur.2021.650653

**Published:** 2021-03-19

**Authors:** Bernard Dan

**Affiliations:** ^1^Université libre de Bruxelles, Brussels, Belgium; ^2^Inkendaal Rehabilitation Hospital, Vlezenbeek, Belgium

**Keywords:** cerebral palsy, bioethics, machine learning, human enhancement, disability, ICF

## Abstract

Current societal and technological changes have added to the ethical issues faced by people with cerebral palsy. These include new representations of disability, and the current International Classification of Functioning, Disability, and Health, changes in legislation and international conventions, as well as applications of possibilities offered by robotics, brain–computer interface devices, muscles and brain stimulation techniques, wearable sensors, artificial intelligence, genetics, and more for diagnostic, therapeutic, or other purposes. These developments have changed the way we approach diagnosis, set goals for intervention, and create new opportunities. This review examines those influences on clinical practice from an ethical perspective and highlights how a principled approach to clinical bioethics can help the clinician to address ethical dilemmas that occur in practice. It also points to implications of those changes on research priorities.

## Introduction

A widely used definition of cerebral palsy describes the condition as a group of permanent disorders of the development of movement and posture, causing activity limitation [as defined within the framework of the International Classification of Functioning, Disability, and Health (ICF), https://apps.who.int/iris/bitstream/handle/10665/42407/9241545429.pdf], which are attributed to non-progressive disturbances that occurred in the developing fetal or infant brain, with the motor disorders being often accompanied by disturbances of sensation, perception, cognition, communication, and behavior, by epilepsy, and by secondary musculoskeletal problems (1). As a complex neurodevelopmental disorder, cerebral palsy presents with a number of ethical challenges across the lifespan. Many of these are in continuing evolution as a result of current societal and cultural changes. Some are general but have a profound impact on individuals with cerebral palsy; others concern them more specifically. Enhanced longevity, for example, is gradually making it obvious that cerebral palsy is not restricted to the pediatric age group [it never was! (2)]. This is the result of improvement in medical management, and it is also calls for attention for specific medical issues (3). In addition, this evolution calls for the reorganization of service in order to adequately address the needs of many adults with cerebral palsy, as well as profound changes in societal perspectives. Societal change has been slow, however, so that a number of issues to which individuals with cerebral palsy are confronted incur ethical dilemmas that often go unrecognized or unaddressed, such as those relating to transition from school age to adulthood (4), the notion of personhood and social participation in adults with developmental disability (5, 6), and aging (7, 8). This leads to disenfranchisement of many individuals with cerebral palsy.

This brief overview examines how technological advances and societal changes influence clinical practice in cerebral palsy from an ethical perspective and highlight how a principled approach to clinical bioethics can help the clinician to address ethical dilemmas that occur in practice. It also points to future research priorities that those changes appear to call for.

## Technological Issues

Dramatic technological advances, some of them potentially empowering, pose ethical questions in cerebral palsy. First of all, access to technological possibilities is typically associated with the risk of a technological imperative that would imply that “if something can be done, then it must be done.” However, it would be simplistic and inappropriate to feel compelled to use any technological possibilities for diagnostic, therapeutic, or other purposes just because they are available. When considering new possibilities, it is decisive to discuss their utility and implications with the patient and family with regard to factors that would be anticipated to interact with the experience of new results. It may be useful to review a few examples and examine how they potentially impact the lives of people with cerebral palsy and even the very concept of cerebral palsy.

New technologies can make diagnosis more accurate than previously. This rekindles ethical issues associated with making diagnoses. Clinicians strive to make diagnoses that will be helpful in improving the person's health and well-being. A diagnosis helps rationalize the observed features and provide prognostic projections. It contributes the person's self-concept, i.e., how one would answer the question “Who am I,” calling for attention in order not to reduce the person to his or her diagnosis and minimizing the risk of stigma. Critically, a diagnosis enables access to appropriate services. The dimension of helpfulness of the diagnosis is more relevant ethically than its accuracy, though the latter contributes to the former. As cerebral palsy is essentially a descriptive diagnosis, the search for causal factors is important, as etiology may have implications for the person and his or her family. Next-generation sequencing is among the technologies that are emerging to their full potential in this respect. Its application to the exome and whole genome has clarified the etiology of cerebral palsy in an increasing number of cases. Development in genetic testing also calls for refinement in the accuracy of the clinical evaluation, challenging the conception that technology is making clinical skills redundant. The results of a recent study illustrate how difficult this remains (10). The study involved pediatric neurologists, rehabilitation physicians, and other movement disorder specialists viewing the same videos of individuals with cerebral palsy in order to identify the presence and evaluate the severity of the core movement disorders that are characteristic of the condition, namely spasticity, dystonia or choreo-athetosis, and ataxia. However, unacceptably high levels of disagreement were found both within and between observers, which suggests that we must very much improve the way we look at and talk about motor features in cerebral palsy if we are to make sense of the diagnostic concept to help patients appropriately. Further progress in genetic evaluation of cerebral palsy also requires a stronger consensus on how cerebral palsy should be defined (11). Insights gained into the understanding of etiological factors, thanks to the results of genetic testing, offer a renewed opportunity to reflect on the usefulness of the construct of cerebral palsy, which to date remains essentially clinical. Based on clinical and research experience, the concept of cerebral palsy remains arguably useful in order to promote development and functioning, and support family well-being in a life-course perspective (2).

Next-generation sequencing has also expanded knowledge about genetic conditions associated with consistent phenotypes, which is relevant to the diagnosis process (9). Additional ethical implications of these development include those that have been explored in genetic counseling. They span across a variety of topics like testing (and carrier testing), predictive value (particularly if testing is performed very early in life), antenatal diagnosis, screening, therapeutic avenues, access to service, and communication.

Information processing of the outcomes of this type of technology increasingly relies on artificial intelligence and machine learning, which depend critically on the quality and relevance of data that are used by algorithms. An example focusing on DNA methylation patterns (not sequencing) showed how this might also be used for predicting the occurrence of cerebral palsy (12). Indeed, artificial intelligence and machine learning support a range of other technological developments for which we can anticipate ethical discussion relating to cerebral palsy (13). In a not-so-distant future, artificial intelligence may allow reliable objective characterization of clinical presentations (14), possibly fed by wearable sensor technology (15, 16). As previously demonstrated in other populations, machine learning can now recognize a range of relevant physical activity behaviors in individuals with cerebral palsy with great accuracy (17). This progress may bring about clinical improvement to patients, but ethical attention will be required to avoid inappropriate surveillance of people (as continuing monitoring is likely to be unnecessary), to avoid excessive normalization (based on the incorrect assumption that it is better for an individual to have recorded values within the normative range), and to solve issues of mediation and substitutions (i.e., the tension between the use of technological devices and human relationships).

Ethical reflection is also required when it comes to addressing challenges to preservation of self-identity that may arise with new advances in robotics and brain–machine interfaces that will increasingly include artificial intelligence. Robotic exoskeletons, neural-control interface devices, transcutaneous electric stimulation of muscles, and brain stimulation techniques are currently being used in research and are starting to be applied in clinical practice. Future refinements of those technologies may prove transformative for users. They could thus amount to providing “human enhancement” in cerebral palsy through applications ranging from functional electric stimulation to patient–robot interaction and neural implants. As with other applications of human enhancement, progress should be considered within a clear ethical frame of reference (18).

Another critical issue with human enhancement, as with all technological advances and indeed all interventions and services, concerns accessibility in a context of social and economic inequality. An even more fundamental ethical question, which is not specific to technology either, is to appreciate to what extent improved functioning effectively leads to improved quality of life. We must accept that it is not necessarily so in cerebral palsy (and other conditions) because the quality of life is essentially based on individual experience, so the assumption that intervention impacts the well-being of a person remains to be proven systematically based on individual situations.

## Societal Issues

Another area of change that comes with ethical questions affects society itself. There has been increasing societal interest for development and children in general (admittedly mostly as consumers in a market economy, with specific ethical problems). The knowledge of cerebral palsy, and even of the term, is still extremely limited in the general population compared to much rarer conditions. It is not entirely clear why; it is possible that opportunities for self-projections offered by communication media play a role, e.g., “this could happen to me” is less of a theme than in the case of acquired conditions, “how would I deal with my own intellect and emotions” may be more appealing in conditions with intact cognitive functioning (which, of course, also occurs in many people with cerebral palsy). Yet, access to information and communication resources has dramatically increased over the last decades, and this may lead to better awareness of cerebral palsy in society. Access to such resources also has ethical implications, and it has contributed to changing the nature of the relationship between health professionals and families, with more ethical implications.

Changing societal perspectives have been incorporated into national legislation in many countries in the last 30 years and in international treaties, most famously the United Nations Convention on the Rights of Persons with Disabilities, which aims to promote and protect the rights and dignity of disabled persons, including respect for the evolving capacities of children with disabilities and respect for their right to preserve their identity, and ensure that disabled persons enjoy full equality under the law. This is important to underline in a context in which legal institutions are often maligned in clinical care, as they actually play a critical role in shaping societal values and can sometimes even offer support for the ethical decision-making. However, legal institutions can also perpetuate bias. Sadly, recent warfare, natural disasters, and health crises including various stages during the COVID-19 pandemic have provided repeated examples of barriers and deprioritization faced by persons with disabilities as a result of political decision and lack of adequate societal response. This is not acceptable; it can, however, be corrected preemptively by raising awareness of the individual and collective human rights to equal access to services and equal treatment of all people with respect and dignity.

Hopefully, this change is underway as there has been a crucial evolution in the cultural perception of disability, which is at long last becoming less negative and more nuanced than in the past. The perspective is gradually moving from an exclusive focus on deficiencies toward functioning, opportunities, and personal expectations. In many settings, the word “handicap” has fallen into disfavor—not all, though, e.g., the term “polyhandicap” has even been coined in France to refer to the condition of people presenting with multiple disabilities that include severe intellectual and motor impairment attributed to the same causal factors (19). The framework of the ICF suggested 20 years ago has been exerting increasing influence on the understanding of health and disability, the setting of goals, and the organization of service. Beyond the opposition between the medical and the social models of disability, the ICF provides a holistic model of functioning and disability that integrates those models together. In contrast with the medical model, which overlooked the role of social forces in enhancing disability, and the social model, which ignored the significance of health problems, the ICF captures a comprehensive range of determinants of disability (20). In addition, the ICF recognizes participation as a specific dimension in one's health condition with direct relevance to one's experience, values, and sense of purpose (21). The ICF follows a universalistic approach that regards disability as a human trait rather than “something that happens to some people” (20). Therefore, it has been recognized to have an intrinsic ethical significance arising from this standpoint and essentially the interactional framework (20). Nevertheless, some pervasive cognitive biases that are still widely prevalent underlie the persistence of a metaphysical model of disability that implies victimization of disabled people (22). This complicates ethical issues. Individuals with those biases tend to have more negative views about people with disabilities and experience more discomfort in dealing with them, contributing to ethical problems relating to social marginalization and exclusion (23).

It is important to recognize cultural disparities and remaining barriers that still limit the disenfranchisement of many disabled individuals worldwide. Socioeconomic factors have been highlighted, including on a global scale. Whereas, 85% of disabled children are estimated to live in low- and middle-income countries (LMIC), <5% of them have access to basic rehabilitative and support services, and they are at higher risk for discrimination, neglect, and abuse. Primary and secondary prevention strategies for cerebral palsy are absent or insufficient (24), and the needs of most children with cerebral palsy, particularly those in rural settings, are not met, as illustrated by low school attendance, care-seeking, and access to assistive devices (25, 26). Lack of stimulation and rehabilitation likely hamper self-care skills, communication, and mobility. Many of those children with cerebral palsy face additional challenges owing to exacerbation of the interplay between disability and culture by poverty, e.g., lack of access to medical services precludes management of pain and epilepsy; and limited knowledge on nutrition compounded by poor food insecurity may result in malnutrition and increased morbidity (26). Local awareness of the prominent importance of functional needs and quality of life has been documented, and action should be taken to support it effectively (27).

## An Ethical Approach

Awareness for the ethical issues associated with cerebral palsy is rising in persons with lived experience, health care professionals, and, to a lesser extent, policy makers and the wider public. It is often considered that an understanding of bioethics is part and parcel of good clinical practice. This can be most evident when dealing with issues directly involving the clinicians' knowledge or skills, partnership with parents or professional team members, communication, and trust. Bioethical practice makes a link between professionalism and humanism, with an emphasis on integrity, humility, respect, empathy, and generosity. Indeed, these attitudes should promote the emergence of ethical decisions in clinical practice, firmly anchored within the interaction with individuals with cerebral palsy and their family. At times, this can be a difficult exercise. As a starting point, it is helpful to realize that while the field of ethics is concerned with questions of morality, a distinction can be made between “common morality” and the ethical reasoning that is at stake here: common morality relates to everyday moral behaviors and decisions that are based on fairly intuitive processes, but what we are concerned with under the banner of ethics aims to resolve perplexing, often difficult moral problems using explicit strategies.

In practical terms, the first step is often to recognize an issue as raising an ethical problem. This may not be obvious if we do not consider the medical, psychological, and social consequences of decision, or if we do not realize that alternative choices are possible. All these choices are potential “solutions” to the ethical problem. It is also useful to consider carefully who are involved in the situation and what their interests are in order to clarify what is at stake and for whom. Ethical questioning and deliberation are the key parts of the exercise and arguably the most important to good clinical practice. They help address challenges and engage in a constructive dialogue with patients and their family, leading to formulating a coherent argument about which of the potential solutions we can reasonably work toward. A number of approaches have been described to that effect. An example is the application of four bioethical principles that were suggested by Beauchamp and Childress (28), as these have proved particularly useful to address ethical dilemmas that occur in the context of cerebral palsy (29). These principles do not impose attitudes or provide ready-made solutions for the resolution of ethical problems, but they can help to organize reflections and support a constructive dialogue. The four principles are respect for autonomy, beneficence, non-maleficence, and justice. When using them, one should rely on an appropriate evidence base. For example, delineating beneficence or non-maleficence or justice in a given situation should not be performed based on intuition or solely on common sense when evidence is available. For some situations, however, the evidence base is currently found wanting. This is in part due to the heterogeneity encountered in cerebral palsy, which has not been comprehensively addressed in research. The condition affects people of all ages and functioning profiles in infinitely different settings across the globe, with much variation in diagnostic and intervention needs. Currently available studies disproportionately include children and young people in Gross Motor Function Classification System levels I to III and often lack controls or long-term outcome measure, resulting in poor quality findings that may be difficult to interpret and generalize. Evaluation of some critical aspects, e.g., economic issues relating to diagnostic and interventional strategies, functional effects in “real life,” and quality of life outcomes, is often lacking. Moreover, owing to their novelty, new approaches have often not lent themselves to useful enough studies. This makes the attraction to new technologies challenging, especially when they are presented with dramatic promises (sometimes even for a cure) by their proponent, and these seem to be supported by anecdotal reports and (social) media hype. The lack of knowledge we have on so many aspects of cerebral palsy calls for particular care in dealing with uncertainty—and points to the importance of promoting good quality research for the benefit of people with cerebral palsy.

The bioethical principle of autonomy describes the patient's right to make his or her own choices. This must be supported by informed consent, which is obtained following person-adapted communication, e.g., avoiding technical jargon. Communication can be very challenging for many people with cerebral palsy (30). This can lead to situations in which the patient's needs remain unmet and hamper his or her autonomy in making decisions. As a consequence, individuals with cerebral palsy can feel that their opinions, perceptions, desires, or rights are neglected (31). An example of topic for which there has been progress but remains much to achieve is reproductive health (32). In many people with cerebral palsy, particularly in children, the right for autonomy is exerted by a third who represents the patients, often the persons who take decisions on their behalf. Parents typically play this role with the assumption that they seek their child's best interest, informed and supported by the professionals (33, 34) (there are rare exceptions when it is questioned whether the decisions they take are in the child's best interest). During adolescence, there is a risk of parental overprotection, which may at times interfere with the normal process of development of individual autonomy and decision-making capacity (31). In this context, health professionals should facilitate the young person's personal autonomy, even when they do not express the need for this.

The principle of beneficence literally means “do good.” In practice, it refers to doing more good than harm. It is not identical to the principle of non-maleficence, which is taken into account separately. The latter is allegedly inspired by the age-old Hippocratic Oath, though the oft-cited (Latin) formula *primum non nocere* meaning “First, do no harm” is not explicitly mentioned in the Oath that suggests that it may be better not to do something, or even to do nothing, than risk causing more harm than good. The principle of non-maleficence also emphasizes the patient's interest, in particular his or her quality of life. A common challenge to both of the beneficence and maleficence principles occurs when caregivers desire to try unproven interventions for cerebral palsy, e.g., hyperbaric oxygen or stem cell therapy (35). One important aspect when addressing this challenge is to make sure they have sufficiently sound information to make informed decisions about risks and benefits. As clinicians, we can also inform them about other interventions as well as opportunities for participating in current or future clinical research—not forgetting the ethical implications of clinical research in children and disabled people, with regard to vulnerability, decision-making, consent, power relationship, and precedence of the patient's inalienable right to the best available management!

The fourth biomedical principle is justice. Contrary to the other three principles, which have a focus on the individual as most of what we are aware that we do in routine clinical practice, the principle of justice concerns the health system in which we organize the management of the patient. It is a complex principle to handle, but it must be considered on an individual basis when addressing ethical dilemmas. The principle of justice implies, for example, fair distribution of care by the health professional as well as by the health system. This raises complex discussions, particularly when it involves management of scarce resources—including in resource-rich settings: how much time should a patient be accorded in a clinic, how much therapy, how do we deal with expensive service or intervention, etc.? With regard to cerebral palsy, two perspectives of justice have been identified, one emphasizing equality among individuals and the other fairness (36). This principle has also been used to guide policies. The principle of justice understood as equality has an individualistic orientation: it sees health as an individual good and cerebral palsy as a problem for the individual, requiring from the health system that it organizes service to address individual needs. The approach is typical for settings entrenched in liberalism, e.g., the United States. The other perspective, which is characteristic of countries that favor a welfare state with a strong social security system, e.g., Belgium, equates this principle with fairness, theorizing health as a collective good, which would imply that cerebral palsy is a problem for the community (including the potential problem of exclusion of individuals from the community), so that the health system should provide service for individuals as members of a community and society as a whole should strive to provide those individuals with fair or equal opportunities. However, comparison of outcomes according to different systems remains difficult to interpret (36).

It should help the developers of future disability policies to let themselves guided by the ethical principle of justice through the challenges they will face as disability policy will become increasingly entangled with other policies, including those that relate to employment and retirement. Employment of people with cerebral palsy in Gross Motor Function Classification System I–III levels without intellectual impairment is currently low compared with the general population (the other groups of people with cerebral palsy are at high risk for unemployment), but it appears to remain stable (37). With the relative increase in less physically demanding jobs, employment can be expected to increase provided enablement is enhanced through vocational interventions aiming at balancing personal capabilities, helping with an ergonomic workplace, and other environmental support. Disability policy in a number of European countries at least is shifting toward social investment in human capital and access to the labor market (38). To remain ethical, this evolution should not be done to the detriment of social protection. Future research should monitor those aspects and evaluate the effect on the well-being of people with cerebral palsy.

## Conclusion

In conclusion, technological and societal changes are associated with some novel ethical issues for people with cerebral palsy, but these can be addressed within the same context of ethical reasoning that is an integral part of ongoing clinical practice. This exercise is entrenched in clinical encounters, in which individual situations may give rise to multiple realities and singular “truths”—and these are not necessarily expected to coincide. Emphasis should be placed on the relevance of a narrative approach that supports a constructive dialogue with the individual with cerebral palsy and the family. Once an ethical dilemma is identified, it can be analyzed using the principles of respect for autonomy, beneficence, non-maleficence, and justice (as both equality and fairness) as guides, bearing in mind that there is no hierarchy of value between these principles, and they do not offer readily available solutions but facilitate a process of shared reflection (29). This approach is mostly pragmatic. It is based on careful observation of signs, facts, and empirical data in a context that is often endowed with incomplete knowledge, requiring conjecture and situational judgment. This approach is not specific to cerebral palsy; it is applied widely in health care. One must recognize that it does not prevent uncertainty from occurring, but the clinician must still aim for the best in every case while running the risk of failure. This type of bioethical reasoning works well to clarify general attitudes with respect to clinical situations, but it is particularly useful for specific decision-making, emphasizing the concept of shared decision-making. Given the enormity of what we do not know, shared decision-making makes allowances for the fact that beneficence and non-maleficence are difficult to assess in many situations. Best practices or guidelines to implement shared decision-making in routine clinical practice with people with cerebral palsy are yet to be developed. Meanwhile, ethical practice remains a process that takes time and may require successive counseling sessions, each demanding appropriate management of current priorities, so the actual resolution of difficult questions should not be expected to be immediate. As in so many areas of cerebral palsy, an increase in the quality of research is necessary. To ensure relevance, future research must involve people with lived experience of cerebral palsy (patients, parents, siblings) when conceiving studies, collecting results, and analyzing findings. Individualized goal setting and realization, well-being, and social determinants are particularly important research priorities, and so are the lifelong and global perspective, not overlooking areas that have been identified as requiring specific attention in low- and middle-income countries, such as ICF domains of activity, participation, and environmental factors, interventions aiming to modify the environment and increasing participation (39). There is a great need for sound qualitative research exploring the perspectives of people with cerebral palsy and for the evaluation of knowledge translation strategies.

## Author Contributions

The author confirms being the sole contributor of this work and has approved it for publication.

## Conflict of Interest

The author declares that the research was conducted in the absence of any commercial or financial relationships that could be construed as a potential conflict of interest.
